# A Novel Mutation in the Stalk Domain of *KIF5A* Causes a Slowly Progressive Atypical Motor Syndrome

**DOI:** 10.3390/jcm8010017

**Published:** 2018-12-22

**Authors:** Massimiliano Filosto, Stefano Cotti Piccinelli, Ilaria Palmieri, Nicola Necchini, Marialuisa Valente, Isabella Zanella, Giorgio Biasiotto, Diego Di Lorenzo, Cristina Cereda, Alessandro Padovani

**Affiliations:** 1 Center for Neuromuscular Diseases, Unit of Neurology, ASST Spedali Civili and University of Brescia, 25100 Brescia, Italy; stefanocottipiccinelli@gmail.com (S.C.P.); nnecchini@gmail.com (N.N.); alessandro.padovani@unibs.it (A.P.); 2 Genomic and Post-Genomic Center, IRCCS Mondino Fundation, 27100 Pavia, Italy; ilaria.palmieri01@universitadipavia.it (I.P.); marialuisa.valente@mondino.it (M.V.); cristina.cereda@mondino.it (C.C.); 3 Department of Molecular and Translational Medicine, University of Brescia, 25100 Brescia, Italy; isabella.zanella@unibs.it (I.Z.); giorgio.biasiotto@unibs.it (G.B.); diego.dilorenzo@yahoo.it (D.D.L.); 4 Clinical Chemistry Laboratory, Diagnostic Department, ASST Spedali Civili di Brescia, 25100 Brescia, Italy

**Keywords:** ALS, hereditary spastic paraplegias, HSP, *KIF5A*, SPG10, axonal neuropathy

## Abstract

*KIF5A* encodes the heavy chain A of kinesin; A motor protein involved in motility functions within neuron. Mutations in the *KIF5A* N-terminal motor domain are known to cause SPG10; An autosomal dominant hereditary spastic paraplegia (HSP), as well as rare Charcot-Marie-Tooth disease 2 (CMT2) cases. Recently C-terminal cargo-binding tail domain mutations have been associated with an amyotrophic lateral sclerosis (ALS) phenotype. Here we describe a subject presenting with an atypical slowly progressive motor syndrome evolving over a period of 4 years; Characterized by walking difficulties; Muscle hypotrophy mainly involving upper limbs and pyramidal signs confined to the lower limbs. Electromyography demonstrated chronic neurogenic damage and active denervation while electroneurography showed slowly worsening axonal damage. We identified the novel heterozygote variant c.2341A>G in the exon 21 of the *KIF5A* gene resulting in the amino acid change p.Lys781Glu. The residue Lys781 is located within the terminal region of the stalk domain and is highly evolutionary conserved. Our findings confirm that mutations in *KIF5A* cause ALS-like phenotypes. However, the stalk domain mutation described here appears to result in an “intermediate” slowly progressive phenotype having aspects resembling ALS as well as HSP and axonal neuropathy. We suggest that *KIF5A* gene should be considered as a candidate gene in all atypical progressive motor syndromes.

## 1. Introduction

Impairment of neuronal transport is recognized as a major target for the development and progression of the neurodegenerative diseases characterized by formation of aggregates i.e., Alzheimer’s disease and Amyotrophic Lateral Sclerosis (ALS) [1,2].

Kinesins are microtubule-associated motor proteins involved in transporting cell organelles, protein complexes, vesicles and in regulating microtubule dynamics in neuronal and non-neuronal cells [1,2]. Specifically, Kinesin-1 is responsible for moving many different types of cargoes in neuronal axons and consists of two heavy chains (KIF5A, KIF5B or KIF5C) and two light chains joined to form a heterotetramer [1,2,3].

The Kinesin Family Member 5A *(KIF5A)* gene encodes for the KIF5A heavy chain of Kinesin-1, which is exclusively expressed in neurons where it is located in cell body, dendrites and axon [3].

KIF5A consists of a three-domain structure: The C-terminal globular tail that is involved in light-chain and cargo binding, the stalk domain, which is an α-helical coiled-coil region involved in heavy chain dimerization, and the N-terminal motor domain which is responsible for the motor activity and hydrolyses ATP in order to bind and move on microtubules [1,2,3]. The motor domain is connected to the stalk domain via a “neck region” which is a mechanical element able to control the direction of motion along a microtubule [1,2,4,5,6].

Mutations in *KIF5A* gene have been identified in association with various motor syndromes such as hereditary spastic paraplegia (HSP) (OMIM #604187) and Charcot-Marie-Tooth disease but also with different phenotypes i.e., neonatal intractable myoclonus (OMIM #617235) [4,5,7,8].

Very recently, whole exome sequencing and genome-wide association studies identify *KIF5A* as a novel ALS gene (OMIM # 617921) [9,10,11].

We reported here clinical and genetic findings of a patient harboring a novel mutation in *KIF5A* who displays a slowly progressive atypical motor syndrome.

## 2. Case Presentation

A 58-year-old Caucasian man complained, since he was 54, of progressive walking difficulties and stiffness at lower limbs, more pronounced on the left side.

Previous clinical history as well as family history was unremarkable.

### 2.1. Evaluation at Onset

The first neurological examination, at age 54, disclosed proximal upper limb (Medical Research Council (MRC) 4/5 on the left side and 3/5 on the right side) and left lower limb weakness (MRC 4/5). Muscle tone was preserved while hypotrophy of the upper limb-girdle, upper limb proximal muscles and left lower limb was present. Winged scapula on the right side was observed. Spontaneous fasciculations were detected in the proximal segments of the upper limbs.

No sensory impairment was reported and cerebellar examination was unremarkable. Tendon reflexes were normal at the upper limbs while knee-jerk and ankle-jerk hyperreflexia was present. No Babinski sign was observed but exhaustible bilateral ankle clone was observed.

No bulbar involvement was present.

Routine laboratory tests (including blood cell count, blood glucose, vitamin B12 and folate, inflammatory parameters) and immunological tests were in the normal range. HBsAg and anti-HCV and anti-HIV antibodies as well as thyroid and parathyroid functions were normal.

Brain and spinal cord imaging was normal as well as cerebrospinal fluid analysis.

Neuropsychological examination showed moderate impairment of executive functions (abstraction, critique, working memory and planning). Abnormal calculation skills were also noted.

Needle electromyography (EMG) showed mild signs of chronic neurogenic damage on quadriceps femoris, tibialis anterior and hand dorsal interosseous muscles. Active denervation was detected in the left tibialis anterior, left quadriceps femoris and right biceps brachii muscles. Electroneurography (ENG) demonstrated a reduction of the compound motor action potential (cMAP) amplitude of the right peroneal nerve.

Motor evoked potentials indicated a hypovolted, unstructured and dispersed cortical motor response with central conduction values increased by derivation of the left lower limb. The contralateral findings were normal.

Somatosensory evoked potential showed bilateral increased central conduction time, more pronounced on the left side.

In a follow-up visit, about one year later, clinical findings were no significantly changed.

### 2.2. Evaluation after Three Years from Onset

Three years after symptom onset, clinical evaluation remained unchanged except for a more pronounced proximal weakness and an increased frequency of fasciculations on upper limbs.

EMG-ENG confirmed a reduction in the cMAP amplitude of the right peroneal nerve (1.20 mV) and demonstrated denervation activity in all the investigated muscles of the lower limbs and in the right arm.

Spirometry showed a forced vital capacity (FVC) of 140%, while maximal inspiratory pressure (MIP) and maximal espiratory pressure (MEP) were reduced to 67.9% and 54%.

### 2.3. Evaluation after Four Years from Onset

**Figure 1:**
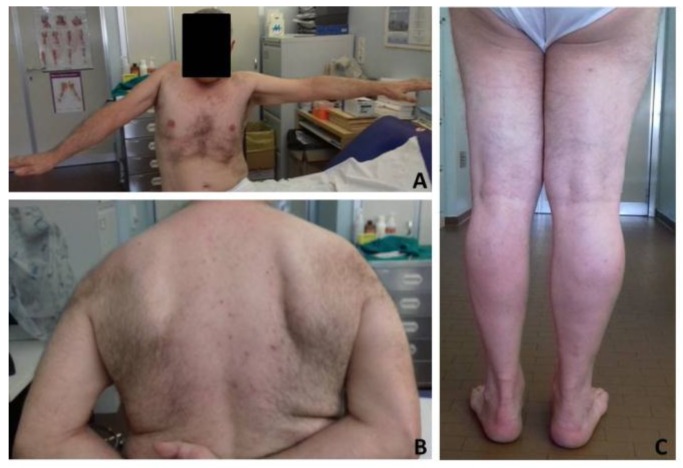
Clinical evaluation after four years from onset. (**A**) Patient presented with upper limb proximal hypotrophy and right upper limb impaired abduction. (**B**,**C**) Unchanged right winged scapula and left lower limb hypotrophy were also observed.

The last neurological examination, four years after symptoms onset, was scarcely changed. Patient exhibited a slightly paretic gait on the right side. He was able to stand from sitting without using his arms as a support. Strength was reduced at the right upper limb which can be abducted only up to 80 degrees (Figure 1A). Mild increase of muscle tone at the lower limbs was detected while distribution of muscle hypotrophy and right winged scapula were unchanged (Figure 1B,C). Sporadic fasciculations were observed at upper limbs, none at lower limbs.

ENG showed reduced amplitude of cMAP of right (1.20 mV) and left (3 mV) peroneal nerves with mild increase in distal latency (6.20 ms on the right side and 4.20 ms on the left side) and normal conduction velocity (43 m/s on both side). Upper limb nerves and both sural nerves were normal.

EMG displayed diffuse denervation activity associated to chronic neurogenic signs.

Right shoulder ultrasound indicated no rupture of muscle tendons.

## 3. Genetic Analysis

After obtaining a written informed consent, DNA was isolated from peripheral blood cells and quantified with NanoDrop ND1000 UV-Vis Spectrophotometer and Qubit^®^ fluorometer (ThermoFisher Scientific, Waltham, MA, USA). Next Generation Sequencing (NGS) analysis (SureSelectQXT Target Enrichment for Illumina Multiplexed Sequencing, Agilent Technologies, Santa Clara, CA, USA) was performed, using a customized panel of 174 genes related to neurodegenerative diseases.

Genes inserted in the customized panel are: *AARS*, *ABCA7*, *ABCD1*, *ACTA1*, *ADH1C*, *ADORA1*, *AGBL5*, *ALS2*, *AMBRA1*, *ANG*, *AP5Z1*, *APEX1*, *APP*, *AR*, *ARHGEF28*, *ASAH1*, *ATL1*, *ATP13A2*, *ATP1A3*, *ATP2B3*, *ATXN1*, *ATXN2*, *ATXN3*, *BAG3*, *BRWD3*, *BSCL2*, *C9ORF72*, *CCS*, *CHCHD10*, *CHCHD2*, *CHMP2B*, *CLN8*, *COQ2*, *CRYM*, *CUL4B*, *CXCL8*, *CYP27A1*, *CYP7B1*, *DAO*, *DCTN1*, *DHTKD1*, *DNAJC12*, *DNAJC6*, *DPP6*, *DTNBP1*, *DYNC1H1*, *EDN1*, *EIF4G1*, *ELOVL7*, *ELP3*, *EPHA4*, *ERBB4*, *EWSR1*, *FA2H*, *FANCL*, *FBXO47*, *FBXO7*, *FEZF2*, *FGGY*, *FIG4*, *FUS*, *GARS*, *GBA*, *GBE1*, *GCH1*, *GDAP1*, *GIGYF2*, *NAT8*, *GLE1*, *GPNMB*, *GRN*, *HEXA*, *HFE*, *HGSNAT*, *HNRNPA1*, *HNRNPA2B1*, *HNRNPA3*, *HSPB3*, *HSPB8*, *ICAM1*, *IGHMBP2*, *IL1A*, *IL1B*, *ITPR2*, *KIF1A*, *KIF5A*, *KIFAP3*, *L1CAM*, *LIF*, *LRRK2*, *METAP2*, *MAPT*, *MATR3*, *MFN2*, *MRS2*, *NAIP*, *NEFH*, *NEK1*, *NOTCH3*, *OPA1*, *OPA3*, *OPTN*, *PANK2*, *PARK2*, *PARK7*, *PDYN*, *PFN1*, *PINK1 PLA2G6*, *PLEKHB2*, *PLP1*, *PNPLA6*, *POLG*, *PON1*, *PON2*, *PON3*, *PRNP*, *PRPH*, *PSEN1*, *PSEN2*, *PTRHD1*, *RAB39B*, *REEP1*, *RNF19A*, *SARM1*, *SETX*, *SCH2*, *SIGMAR1*, *SLC1A2*, *SLC1A4*, *SLC2A1*, *SLC52A1*, *SLC52A2*, *SLC52A3*, *SMN1*, *SNCA*, *SOD1*, *SORL1*, *SPAST*, *SPG11*, *SPG21*, *SPG7*, *TACR1*, *SPTLC1*, *SQSTM1*, *SS18L1*, *SYNE1*, *SYNJ1*, *TAF1*, *TAF15*, *TARDBP*, *TBK1*, *TNF*, *TOMM40*, *TREM2*, *TRIM28*, *TRPV4*, *TUBA4A*, *TUBB4A*, *UBA1*, *UBE2A*, *UBE3A*, *UBQLN2*, *UNC13A*, *UCHL1*, *VAPB*, *VCP*, *VEGFA*, *VPS13A*, *VPS13C*, *VPS35*, *VPS54*, *ZFYVE26*, *ZHX2*.

FastQ files generation was performed using MiniSeq provided software (Real Time Analysis RTA v.1.18.54 and Casava v.1.8.2, Illumina, Inc., San Diego, CA, USA). FastQ files provided for each sample, containing mate paired-end reads after demultiplexing, were trimmed for adapter removal with cutadapt (v1.10).

Trimmed FastQ files were aligned to hg19 reference genome exploiting the Burrows-Wheeler transformation-based alignment via BWA-mem software v7.5a [12]. BAM files were sorted and indexed via samtools v1.19 and Picard-tools v1.95 (http://broadinstitute.github.io/picard/). GATK V3.1 was used for insertions/deletions realignment (with RealignTargetCreator, IndelRealigner and BaseRecalibrator) and variant calling (with UnifiedGenotyper) according to GATK Best Practices recommendations [13,14,15]. Produced VCF were processed with eVAI software (enGenome, Pavia, Italy) for annotation and variant classification.

**Figure 2:**
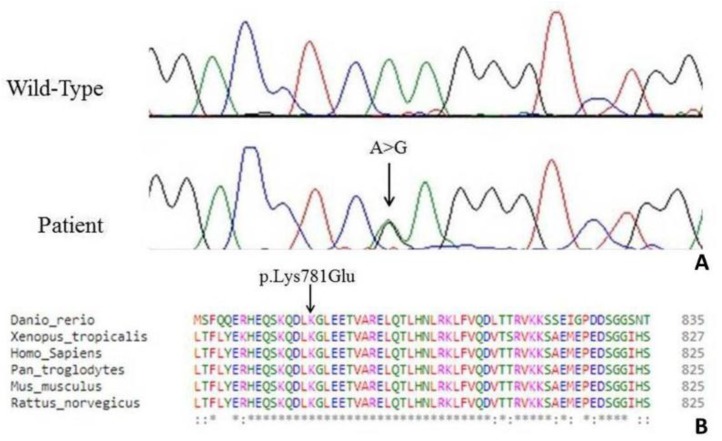
(**A**) Electropherogram showing the novel heterozygote variant c.2341A>G in the exon 21 in the patient compared to a control sequence. (**B**) A partial alignment of *KIF5A* from different species showing the residue Lys781 located within the terminal region of the stalk domain and highly evolutionary conserved.

Through NGS analysis we identified the novel heterozygote variant g.32690A>G, c.2341A>G in the exon 21 of the KIF5A gene resulting in the amino acid change p.Lys781Glu (Figure 2A).

In silico prediction software, such as Polyphen2 (http://genetics.bwh.harvard.edu/pph2/) and Mutation Taster (http://www.mutationtaster.org/), defined this variant as damaging or disease causing respectively. The c.2341A>G mutation was then confirmed by Sanger sequencing on ABI 3130xl Genetic Analyzer (Applied Biosystems, Foster City, CA, USA). The forward and reverse primers were 5′GAGGCAGGAGGAAGGAGAGT3′ and 5′AGAAATGAAGCCTCCCCACT3′ respectively (Metabion International AG, Planegg/Steinkirchen, Germany) and PCR cycling conditions were 95 °C for 4 min, followed by 38 cycles of 95 °C for 30 s, 59 °C for 30 s, 72 °C for 30 s, with a final extension step of 7 min at 72 °C.

Mutation nomenclature is based on RefSeq NM_004984.2 (GenBank) (considering the A of the ATG as nt 1) and on RefSeq NG_008155.1 for *KIF5A* cDNA and genomic sequences respectively, and follows the guidelines of the Human Genome Variation Society (http://www.hgvs.org/mutnomen/).

The residue Lys781 is located within the terminal region of the stalk domain and is highly conserved (Figure 2B).

**Figure 3:**
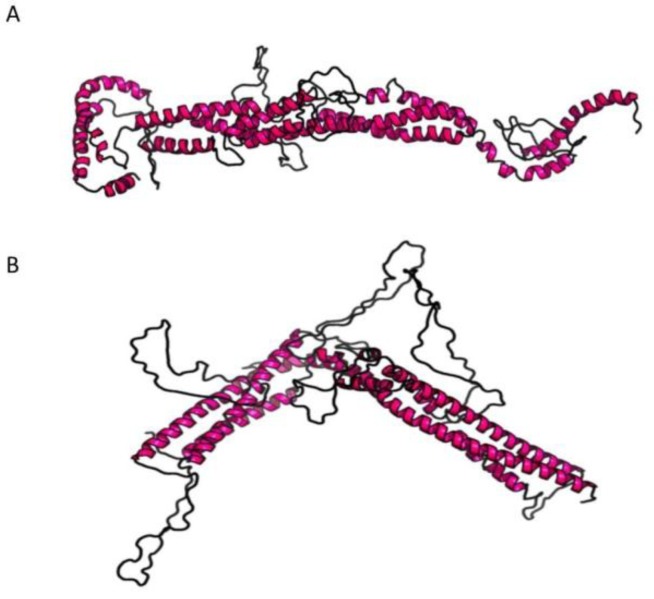
Image of the predicted 3D structure of the stalk domain of protein KIF5A. (**A**) Wild-type structure of the stalk domain; (**B**) Mutated structure of the stalk domain.

For KIF5A, only the three-dimensional (3D) structure of the kinesin motor domain has been well characterized [16], while no structural information have been deposited in the Protein Data Bank (PDB) for the stalk domain yet. Thus, the molecular modelling prediction was done with a software able to predict the 3D structure only on the bases of the amino acid sequence. RaptorX (http://raptorx.uchicago.edu/) software was employed to predict the 3D structure of the stalk domain, querying the portion of the protein from amino acid 331 to 906. The 3D wild-type structure predicted (*p*-value 7.14 × 10^−5^) reveals that the stalk domain is mainly formed by coiled alpha-helices structures (Figure 3A). The same was done to see how the 3D structure changed upon insertion of the Lys781Glu mutation (Figure 3B). As shown, the 3D structure of the stalk domain containing the mutation is highly altered compared to the wild-type (Figure 3B; *p*-value 7.98 × 10^−5^), reinforcing the pathogenic role of the Lys781Glu mutation.

**Figure 4:**
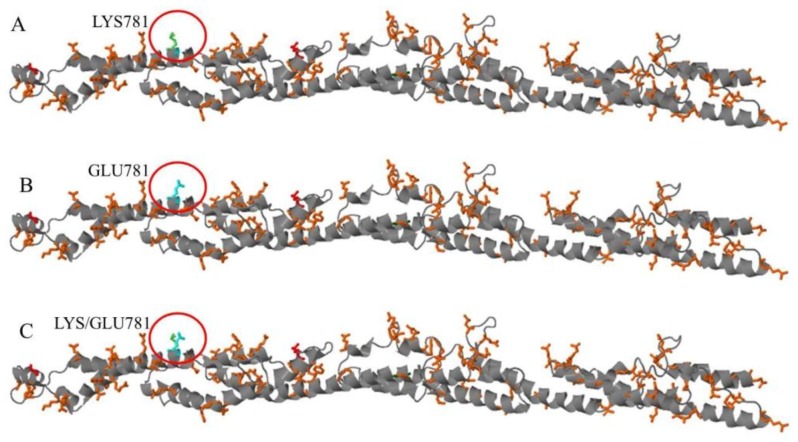
Image of the coiled coil region from amino acid 331 to amino acid 906 of the KIF5A protein and the spatial visualization of the wild-type and mutant amino acids at targeted position 781. (**A**) Spatial visualization of the Lysine at position 781; (**B**) Spatial visualization of the Glutamate at position 781; (**C**) Spatial visualization of both the Lysine and the Glutamate at position 781.

The stalk domain of the KIF5A protein, involved in heavy chain dimerization, goes from amino acid 331 to amino acid 906 [10]. This domain is characterized by a coiled coil region, where two or more alpha-helices are coiled together. In a coiled coil region the hydrophobic amino acids are involved in the stability of the coiled structure, while charged amino acids are usually exposed to the surface, where they are in contact with water molecules or are involved in salt bridges important for the stabilization of the 3D structure or for the dimerization with other proteins. The Lysine 781 variation (Figure 4A) is a positively charged polar amino acid that changes into a Glutamate, which instead is a negatively charged polar amino acid (Figure 4B). Moreover, based on Genomic position to 3D data (G23D, https://www.sheba-cancer.org.il/cgi-bin/variants/G23D.cgi) software, these two amino acids present a different spatial orientation (Figure 4C) that, together with the different electric charge, may alter both the interaction among alpha-helices in the coiled coil domain and the dimerization of the heavy chain.

## 4. Discussion

Autosomal dominant hereditary spastic paraplegia type 10 (SPG10) and Charcot-Marie-Tooth disease type 2 (CMT2) are the main phenotype associated to *KIF5A* mutation [5,17,18,19,20,21].

Quite recently, *KIF5A* has been identified as a novel gene associated with ALS [9,10].

Interestingly, mutations in different *KIF5A* domains seem to cause different phenotypes.

Missense mutations in the N-terminal motor domain are mainly linked to SPG10 and CMT2 [10]. They are clustered in two areas of motor domain, the switch regions SWI (199–204) and SWII (232–237), that are necessary for microtubules interaction.

Differently, ALS-associated mutations are predominantly located at the C-terminal cargo-binding tail domain [9,10]. The highly penetrant ALS mutations are loss-of-function changes and the affected patients display younger age at onset and longer survival respect to the typical ALS subjects [10]. No variants previously associated with SPG10 or CMT2 was detected in ALS patients [10].

Beyond the three main phenotypes associated with *KIF5A* mutations, more complex and overlapping phenotypes are emerging and it is now evident that mutations in this gene may account for a clinically heterogeneous spectrum of diseases [4,5,22,23,24,25,26]. This was expected because *KIF5A* is a pan-neuronal expressed gene and, since mutations affect axonal transport, all the neurons with long axons (i.e., those of corticospinal tracts and peripheral nerves) are likely to be involved in the pathological process [25].

Especially the simultaneous involvement of central and peripheral pathways is notable. Complicated *KIF5A*-linked HSP phenotypes displaying axonal neuropathy, parkinsonism, mental retardation and retinitis pigmentosa were recently described [4,5,22,23,24,25].

In a previous study, we identified a novel variant located at the C-terminus of the *KIF5A* motor domain in a patient with a HSP/axonal neuropathy phenotype and therefore confirmed that the “mixed” central-peripheral involvement is a frequent SPG10-related clinical picture [27].

Here we report on a patient which developed, over a period of at least four years, a slowly progressive motor syndrome mainly involving proximal segments of limbs in an asymmetrical way. The patient presented signs of upper and lower motor neuron involvement, with the first ones more severe at lower limbs (pyramidal abnormalities and ankle clone) and the second ones at upper limbs (muscle hypotrophy, fasciculations).

In this patient, slow evolution time and survival are in line with what has already been reported in *KIF5A*-linked ALS patients in which an increased disease duration compared to the median survival time of classical ALS patients (20–36 months) was described [28]. At the same way, age of onset is in the range of the other *KIF5A*-related ALS cases (median age of onset at 46.5 years) [10].

However, two interesting findings deserve attention. Firstly, pyramidal signs remained confined to the lower limbs all over the time as in the HSP and, secondly, ENG signs of an axonal damage of lower limb motor nerves developed in the course of disease.

For this reason, this clinical picture could be considered an “intermediate form” of *KIF5A*-linked motor syndrome, having clinical and electrophysiological aspects resembling ALS, HSP and mild axonal neuropathy.

The variant here identified, p.Lys781Glu, is located within the terminal region of the stalk domain, outside from the motor domain which is a hot spot for HSP pathogenic mutations as well as from the cargo binding domain in which loss of function changes involved in ALS phenotype are located.

Few other mutations outside of motor and cargo domains were described, falling in the stalk domains or in the neck region and they are associated with a HSP phenotype [18,20].

Therefore, our patient represents the first case, albeit atypical, of ALS phenotype associated to a stalk domain mutation.

How mutations in the same gene affect transport activity either selectively in upper motor neurons and in peripheral axons or simultaneously in both central and peripheral motor pathways remain unclear.

Probably, the KIF5A-mediated transport mechanisms presuppose a functional complexity for this protein higher than expected [17]. In fact, both anterograde and retrograde types of neurofilament transport are involved in *KIF5A*N256S mutant mouse neuronal cells [29,30].

Since missense mutations within motor domain cause reduce transport velocity by affecting microtubule binding and ATP hydrolysis, the resulting defective anterograde transport of cargo and disarray of neuronal axoplasmic flow should lead to the axonal retrograde degeneration observed both in HSP and CMT2 [4,18,20,31].

Differently, the loss-of-function variants within the C-terminal domain, by disrupting binding with cargo proteins, could lead to protein accumulation and pathological aggregation within the cell body resulting in a primary motor neuron damage [10].

Stalk domain is a long alpha-helical coiled coil domain and is connected via a flexible neck linker to the motor domain and, on the other side, to the terminal tail domain which associates with the light-chains [2,6,32]. The stalks of two heavy chains intertwine to form the coiled coil that directs the heavy chain dimerization, a process necessary to bind two light chain and form the complete protein [3,33,34].

Hypothetically, some stalk domain mutations could interfere with the correct structure of the protein and affect both motor and binding functions, therefore providing a possible explanation for the overlapping phenotype seen in our patient.

In any case, whatever the underlying mechanism, our study confirms that *KIF5A* mutations may be linked to slowly progressive and early-onset ALS phenotypes. Also changes in stalk domain can be associated to ALS, but the resulting phenotype seems to suggest a more complex overlapping motor syndrome.

Therefore, *KIF5A* gene should be a candidate gene in all the cases of atypical progressive motor syndromes.
